# *Ehrlichia* spp. infection worsens cardiac damage in dogs with canine visceral leishmaniasis

**DOI:** 10.1590/S1984-29612024023

**Published:** 2024-05-31

**Authors:** Leticia Gomes Zanfagnini, Janildo Ludolf Reis, Vinícius Novaes Rocha, Soraia Figueiredo Souza, Karina Yukie Hitara, Mary Marcondes, Acácio Duarte Pacheco

**Affiliations:** 1 Universidade Federal do Acre – UFAC, Rio Branco, AC, Brasil; 2 Departamento de Medicina Veterinária, Universidade Federal de Juiz de Fora – UFJF, Juiz de Fora, MG, Brasil; 3 Universidade Estadual Paulista – UNESP, Araçatuba, SP, Brasil

**Keywords:** Coinfection, dog disease, heart disease, hemoparasitosis, Leishmania chagasi, Coinfecção, doenças de cão, cardiopatia, hemoparasitose, Leishmania chagasi

## Abstract

In endemic areas for canine visceral leishmaniasis (CVL), the occurrence of coinfection with other pathogens, such as *Ehrlichia* spp., has been associated with worsening of the clinical condition. The study aimed to evaluate the occurrence of histological changes in the myocardia of dogs naturally infected with *Leishmania chagasi* with or without coinfection with *Ehrlichia* spp.. We evaluated paraffin-embedded myocardial sections from 31 dogs, affected by either *L. chagasi* alone or coinfected with *L. chagasi* and *Ehrlichia* spp., to compare the extent and degree of cardiac damage. The blocks were divided into two groups. G1 (dogs infected only by *L. chagasi)* and G2 (dogs coinfected with *L. chagasi* and *Ehrlichia* spp.). The right atrium free wall, right ventricle free wall, left ventricle, and interventricular septum of all groups were evaluated. Cardiac alterations were observed in 41.93% (52/124) of the fragments evaluated and inflammatory infiltrate was the most common pattern found. The G2 group showed a higher incidence of myocarditis, with 61.53% (32/52), compared to the G1 group, in which 20 out of 72 cases (27.7%) exhibited histopathological changes (p <0.05). These findings confirmed that coinfection can potentiate cardiac damage in dogs.

## Resumo

Em áreas endêmicas para leishmaniose visceral canina, a ocorrência de coinfecção com outros patógenos, como *Ehrlichia* spp., tem sido associada à piora do quadro clínico dos animais. O presente estudo teve como objetivo avaliar a ocorrência de alterações histológicas no miocárdio de cães naturalmente infectados por *Leishmania chagasi,* associadas ou não à ocorrência de coinfecção por *Ehrlichia* spp.. Para isso, foram avaliados fragmentos parafinizados de 31 cães afetados por *L. chagasi* e *Ehrlichia* spp. Para a avaliação e a comparação dos danos cardíacos, os blocos foram divididos em dois grupos, sendo o G1 (infecção somente por *L. chagasi*) e o G2 (animais coinfectados com *L. chagasi* e *Ehrlichia* spp.). Foram avaliados a parede livre do átrio direito, parede livre do ventrículo direito, ventrículo esquerdo e septo interventricular de todos os grupos. Alterações cardíacas foram observadas em 41,93% (52/124) dos fragmentos avaliados, sendo o infiltrado inflamatório o tipo mais comum de lesão encontrada. O G2 apresentou uma incidência superior de miocardite, com 61,53% (32/52), enquanto no G1, em 27,7% (20/72), foi possível observar a ocorrência de miocardite (p <0,05). Esses achados confirmam que a coinfecção potencializa o dano cardíaco em cães.

## Introduction

Canine visceral leishmaniasis (CVL) is associated with a variety of clinical signs that directly relate to the immune mechanisms triggered and organs affected at the time of infection, making it difficult to diagnose. Progressive weight loss, hepatosplenomegaly, lymphadenomegaly, muscle atrophy, anemia, and skin lesions are common symptoms, as well as ophthalmic, digestive, neurological, and renal alterations. The latter is usually associated with a deterioration of the patient's clinical condition and can lead to death (Baneth et al., 2008; Melo et al., 2009).

Cardiac damage in dogs with CVL has been reported, despite the absence of obvious clinical signs (Rosa et al., 2014; Costagliola et al., 2016). Studies have been performed to evaluate the mechanisms that induce these changes in canine species (Torrent et al., 2005; López-Peña et al., 2009). Although myocardial alterations have been confirmed, and the parasite has been identified, the mechanism remains poorly understood (Andrade et al., 2014; Costagliola et al., 2016).

In endemic regions for CVL, the occurrence of co-infection with other pathogens, such as *E. canis, A. platys, Hepatozoon* spp., and *Babesia* spp. has been observed and associated with clinical worsening (Paulan et al., 2013; Attipa et al., 2018; Baxarias et al., 2018). Histological changes in the brains of dogs with CVL have been shown, and the occurrence of co-infection between *L. chagasi* and *Ehrlichia* spp*.* has been identified as a factor associated with the worsening of these conditions (Oliveira et al., 2017; Attipa et al., 2018). Similarly, in the central nervous system, a greater intensity of the inflammatory infiltrate was described in dogs infected with *E. canis* and *T. gondii*, compared to animals infected with CVL only, corroborating the association between pathogens that could intensify the clinical signs and evoke further histological changes (Oliveira et al., 2017).

We considered that the presence of coinfection in dogs with CVL and *Ehrlichia* spp*.* could enhance cardiac damage, since both diseases have the ability to develop myocardial lesions in infected patients (Diniz et al., 2008; Rosa et al., 2014; Costagliola et al., 2016). To date, there are no studies evaluating the occurrence of worsening of cardiac damage in dogs coinfected with *L. chagasi* and *Ehrlichia* spp*.* Therefore, the present study aimed to compare the hearts of dogs coinfected with *L. infantum* and *E. canis* with those of dogs infected only with CVL, in order to understand the mechanism by which *Ehrlichia* spp. promotes this worsening of the lesion.

## Materials and Methods

The samples consisted of 124 archived myocardial fragments, fixed in formalin and embedded in paraffin, from 31 dogs age over six months, regardless of sex and race, naturally affected by *Leishmania chagasi* e *Ehrlichia* spp.

The diagnosis of CVL was based on direct parasitological examination, lymph node aspiration or bone marrow cytology, to confirm the presence of parasite amastigote forms. SNAP 4Dx Plus (IDEXX Laboratories Inc.) tests were performed using the manufacturer’s instructions, to identify *E. canis/E. ewingii* antibodies. All dogs were considered symptomatic for CVL, although none had clinical signs of cardiac disease, alterations in rhythm or pulse, presence of murmur, or hypophonesis of heart sounds.

To evaluate myocardial lesions, the blocks were divided into two groups. G1 (n=18) was composed of dogs with CVL, while G2 (n=13) encompassed dogs coinfected with *Leishmania chagasi* and *Ehrlichia* spp. Four fragments of the cardiac muscle of these animals were evaluated: right atrium free wall (RA), right ventricle free wall (RV), left ventricle free wall (LV), and interventricular septum (IVS), for a total of 124 fragments.

Histological sections of 5 micrometers thick myocardium were stained with hematoxylin and eosin (HE) and picrosirius red (PS) (Tolosa et al., 2003). In the H&E-stained sections, the morphological aspects of the cardiac tissue were evaluated, such as the presence of degenerate fibers, and the location (epicardium, myocardium, and endocardium), intensity, and distribution (focal or multifocal) of the inflammatory infiltrate. In order to assess the areas of myocardial fibrosis, the sections were subjected to PS, which stains the collagen fibers.

The heart sections of dogs with CVL were compared with those of animals coinfected with *L. chagasi* and *Ehrlichia* spp*.* The lesions were semi-quantitatively scored on a scale by one experienced histopathologist for the presence and intensity of changes (Rosa et al., 2014; Casamián-Sorrosal et al., 2021) of 0 to 4: 0 = absent, 1 = minimum (scant inflammatory cells scattered with no tissue disruption), 2 = mild (few inflammatory cells arranged in small foci with no tissue effect), 3 = moderate (inflammatory cells arranged in larger foci, or multifocal to coalescent arrangement with mild to moderate local tissue disruption), and 4 = severe (larger areas of inflammatory infiltration in multifocal to coalescent arrangement).

Results were compared using the Mann Whitney test, and lesion intensity in the different cardiac areas was compared using the Friedman test. Statistical p-value <0.05 was set at significant. All Statistical analysis was performed using a commercially available software program GraphPad Software, San Diego, California, USA.

## Results

**Figure 1:**
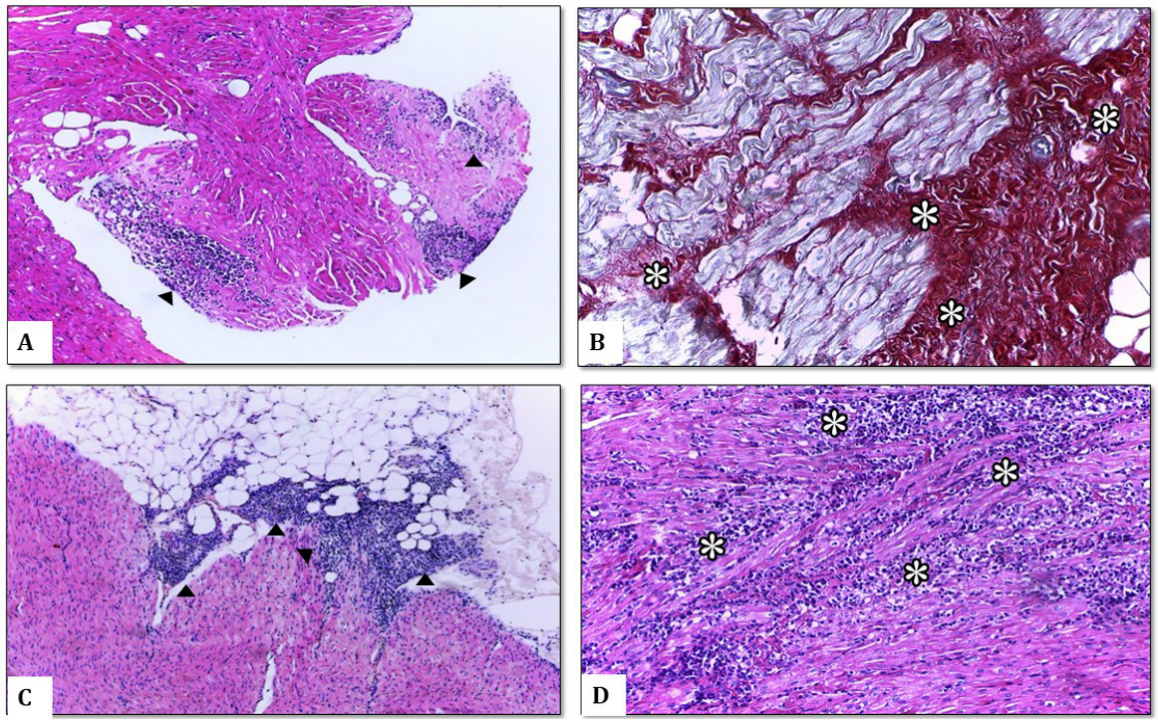
A: Arrowos- Multifocal inflammatory infiltrate in a dog's right atrium. Hematoxylin and eosin (HE, 100x). B: Asterisks-Large area of ​​fibrosis staining in the right atrium. Picrossirius Red (PS, 400x). C: Arrowos-Left ventricle; dog. Inflammatory infiltrate in the epicardial and myocardial region associated with leishmaniasis. Insertion: mononuclear cells predominate. Hematoxylin and eosin (HE,100x). D: Asterisks- Intense lymphoplasmocytic infiltrate in a dog's right atrium. (HE, 400x).

Heart lesions were present in 52/124 (41.93%) of the examined sections. In G1, they were represented in 20/72 (27.7%) of the samples, and in G2 lesions were noted in 32/52 (61.53%) of the sections. Comparing G1 and G2, there was a significant increase (p=0.034) in the severity of lesions in G2. The heart changes were mainly characterized by lymphoplasmacytic and histiocytic infiltration and were observed in all samples that presented some degree of injury (Figure 1A).

Fibrosis was more frequently observed in G2 myocardial fragments, occurring in six of these fragments. In G1, however, it was possible to observe fibrosis in only two fragments of the total evaluated (Figure 1B). No other histopathological findings, such as myocardial degeneration or necrosis, were observed in all evaluated sections.

The identification of amastigotes forms of *Leishmania* spp. in the myocardium occurred in a single animal, belonging to G1. The parasite was identified along with the inflammatory infiltrate in the right atrium. In the other fragments, amastigotes forms were not visualized, even when inflammatory infiltrates were present.

There was a higher incidence of myocarditis 38/52 (73.0%) of the total. The endocardium (7.69%, 4/52) and epicardium (1.92%, 1/52) also presented inflammation, but at lesser degrees. There was an intense amount of inflammatory infiltrate simultaneously in the different layers of the heart (epicardium, myocardium, and endocardium) in 7.69% (4/52) of the samples (Figure 1C).

The RV was the region most affected, with 14/31 (45.1%) of the fragments showing some degree of inflammation, followed by the RA and IVS (41.9%, 13/31 in total). The LV showed some degree of injury in 12/31 (38.70%) of the fragments. The occurrence and intensity of inflammation showed a significant difference only in the IVS between groups (p=0.034). The inflammation intensity ranged from mild to intense; however, the occurrence of moderate (Figure 1D) alteration was more prevalent, corresponding to 16/52 (30.7%) of the total. Although the intensity of inflammation was higher in the RV than in other regions, the difference was not significant (p=0.034).

**Figure 2:**
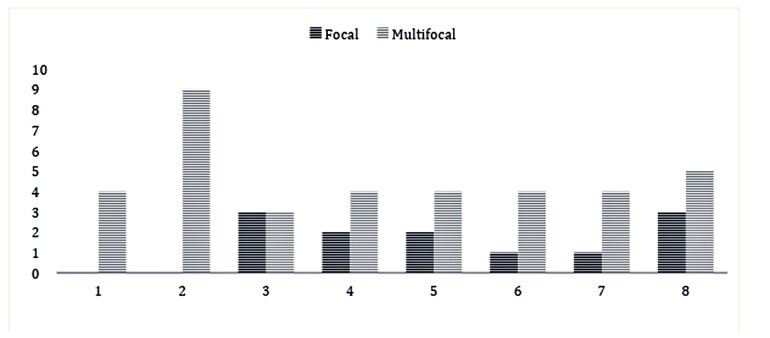
Distribution of lesions according to the region of the myocardium evaluated in groups G1 and G2.

The distribution of the inflammatory infiltrate occurred in a multifocal way in most of the evaluated tissues, mainly in the G2 group. Although less frequent, focal lesions were also observed. The distribution of lesions according to the assessed myocardial region is exemplified in Figure 2.

The prevalence and severity of the main histologic changes are summarized in Table 1.

**Table 1:** Intensity of inflammatory infiltrate in the right atrium free wall (RA), right ventricle free wall (RV), interventricular septum (IVS) and left ventricle free wall (LV) of the myocardium of 31 dogs naturally affected by CanL without coinfection (G1) and with coinfection (G2) by *E. canis*. Absolute number (N) and percentage of fragments evaluated (%).

Classification	RA				RV				LV				IVS			
	G1		G2		G1		G2		G1		G2		G1		G2	
	N	%	N	%	N	%	N	%	N	%	N	%	N	%	N	%
Absent	14	77.7	4	30.7	12	66.6	5	38.4	12	66.6	7	53.8	13	72.2	5	38.4
Slight	-	-	3	23.0	2	11.1	-	-	4	22.2	3	23	3	16.6	2	15.3
Mild	-	-	4	30.7	1	5,5	5	38,4	-	-	1	7.6	1	5.5	4	30.7
Moderated	3	16.6	2	15.3	3	16.6	3	23	2	11.1	1	7.6	1	5.5	1	7.6
Severe	1	5.5	-	-	-	-	-	-	-	-	1	7.6	-	-	1	7.6

## Discussion

In the present study we confirmed an increase in the frequency and severity of heart lesions in animals coinfected with *Leishmania chagasi* e *Ehrlichia* spp. Cardiac lesions in dogs with CVL have been frequently reported. However, even though 80% of dogs in endemic regions can be coinfected with *L. chagasi* and *Ehrlichia* spp. (Baxarias et al., 2018), the histopathology of the heart has not been described in these animals yet.

These results corroborate with previous clinical studies in dogs with CVL coinfected with other pathogens such as *E. canis*, *A. platys*, *Hepatozoon* spp., and *Babesia* spp., in which there was worsening of the clinical condition due to coinfection (Paulan et al., 2013; Baxarias et al., 2018; Attipa et al., 2019). Despite that, the mechanism of exacerbation of the clinical picture in co-infected animals is not fully understood.

In our study, due to the natural infection context, we cannot assert which pathogen was the primary one. However, it is known that it can act as a facilitating agent for the establishment of secondary organisms (Otranto et al., 2009; Attipa et al., 2019; Apostolidis et al., 2023), and studies have shown that dogs with CVL are likely to be additionally infected with *Ehrlichia* spp. and other agents (Baxarias et al., 2018). Still in this sense, since immunohistochemistry for Leishmania spp. was not performed, it was not possible to determine the role of the parasite in myocardial injury.

Infection by *L. chagasi* can lead to a failure in the host's cellular and humoral immune response, contributing to the establishment or reactivation of a pre-existing infection by *Ehrlichia* spp. (Barbiéri, 2006; Mekuzas et al., 2009). Additionally, *E. canis* can cause a reduction in MHC II receptors, compromising the presentation of T-CD4 lymphocytes and facilitating the progression of CVL (Harrus et al., 2003), further suggesting that certain comorbidities may be precipitating factors for the transition from the subclinical infection by *L. chagasi* to the overt CVL (Apostolidis et al., 2023).

In animals coinfected with *L. chagasi* and *Ehrlichia* spp., heart inflammation was more severe and the lymphoplasmacytic myocarditis was the mainly pattern found in these animals, confirming the findings of other studies that identified the worsening of lesions in different organs as a result of coinfection (Mekuzas et al., 2009; Rosa et al., 2014; Attipa et al., 2019;). Previous studies reported lymphoplasmacytic myocarditis ranging from mild to moderate in symptomatic and asymptomatic dogs (Alves et al., 2010; Rosa et al., 2014; Costagliola et al., 2016), corroborating our findings, with the same pattern of intensity in dogs only with CVL. In the present study, none of the analyzed sections showed degeneration or necrosis. Due to the use of samples from naturally infected animals, we were unable to control the collection of samples at different time points post-infection.

We did not find a correlation between the extent and arrangement of the inflammatory infiltrate, semi-quantitatively scored from 0 to 4, with the clinical symptoms of the animals. Despite histopathological findings revealing a greater extent of myocarditis in coinfected dogs, these findings were not sufficient for the clinical manifestation of cardiopathy in these animals, such as the occurrence of murmurs, arrhythmias, and alterations in arterial pulse (Diniz et al., 2008). We believe that this may be related to the asymptomatic nature of both pathologies or compensatory mechanisms, which may help maintain blood pressure and blood flow, temporarily masking clinical signs (Van Amstel et al., 1987; Diniz et al., 2008). This highlights the difficulty in identifying heart diseases in infected dogs (Zabala et al., 2005; Godoy et al., 2016).

The results of the study conducted by Diniz et al. (2008) indicate that cardiomyocyte injury occurs in dogs naturally infected by *E. canis*. Additionally, dogs with acute *E. canis* infection showed a higher risk of myocardial cell injury compared to other diseased dogs. The myocardial depressant factor was described in myocarditis induced by the systemic inflammatory response syndrome (SIRS) in mice and dogs, involving nitric oxide, arachidonic acid derivatives, endotoxin, cytokines, and reactive oxygen species (Diniz et al., 2008). In the study conducted by Gianfranchesco Filippi et al. (2019), it was observed that animals affected by chronic Ehrlichiosis presented arrhythmic events, but these arrhythmias were not associated with a worse prognosis.Thus, we believe this could be a possible explanation for the greater manifestation of myocardial injury observed in Group 2 (G2).

The occurrence of cardiac clinical signs in dogs with CVL has already been described; however, they are generally not considered in practice (Rosa et al., 2014). In these cases, changes can only be confirmed by the identification of microscopic alterations in the myocardium, which are independent of laboratory, electrocardiographic, and clinical findings, alerting us to the difficulty of identification of heart disease in infected dogs (Zabala et al., 2005; Godoy et al., 2016).

Our results are similar to those previously described, with regard to the right side of the heart being the most affected region in dogs naturally affected by *L. chagasi* (Rosa et al., 2014; Costagliola et al., 2016). The pathogenesis of perivascular and interstitial cardiac fibrosis involves the response to hormonal stimuli, primarily related to the renin-angiotensin-aldosterone system and hemodynamic stimulation, specifically increased blood pressure and inflammatory mechanisms (Nicoletti et al., 1995). Hypertension is invariably associated with the infiltration of inflammatory cells within large vessels (Nicoletti et al., 1995). During the inflammation process occurring in the pressure-overloaded heart, macrophages and T lymphocytes can release cytokines to counteract the effects on cardiac cells (Nicoletti & Michel, 1999). The metabolism and proliferation of fibroblasts and cardiac myocytes, as well as the renewal of the extracellular matrix, provide targets for these molecules. However, cytokines can also exert feedback to amplify or inhibit the inflammatory response itself, due to their chemotactic and/or anti-inflammatory properties (Hinglais et al., 1994; Krawczak et al., 2015).

The findings of the present study demonstrate that, although there are evident lesions in the myocardium of dogs infected with *Leishmania chagasi* and an exacerbation of the intensity and extension of the inflammatory infiltrate in animals presenting coinfection between *Leishmania chagasi* and *E. canis*, clinical signs may be absent. However, it is reasonable to assume that, in the long term, animals may exhibit clinical signs of heart disease, especially in coinfected animals. Nevertheless, further experimental studies are needed to better elucidate the relationship and understand more comprehensively how *Ehrlichia* spp. could contribute to the exacerbation of lesions caused by *Leishmania* spp.

## Conclusion

In conclusion, our findings support the evidence that co-infection with *Leishmania* *chagasi* and *Ehrlichia* spp. leads to exacerbation of cardiac damage in dogs. However, more studies are needed with greater control over the period of infection, in order to better clarify the mechanism of exacerbation of inflammation. Therefore, in regions endemic for both agents, it is essential that early identification of cardiac changes is investigated in order to avoid rapid clinical progression.
